# Near-Compensated Ferrimagnetism in Disordered Co_0.5_Mn_1.5_Al Half-Heusler Alloy: Experimental and Theoretical Studies

**DOI:** 10.3390/ma18194449

**Published:** 2025-09-23

**Authors:** Emese Bender, Răzvan Hirian, Cristian Leoştean, Roman Atanasov, Radu George Haţegan, Lucian Barbu-Tudoran, Diana Benea

**Affiliations:** 1 Faculty of Physics, Babeş-Bolyai University Cluj-Napoca, Kogãlniceanu Str 1, 400084 Cluj-Napoca, Romania; emese.bender@stud.ubbcluj.ro (E.B.); razvan.hirian@ubbcluj.ro (R.H.); atanasov.roman@ubbcluj.ro (R.A.); radu.hategan@ubbcluj.ro (R.G.H.); 2 National Institute for Research and Development of Isotopic and Molecular Technologies, 400293 Cluj-Napoca, Romania; cristian.leostean@itim-cj.ro (C.L.); lucian.barbu@itim-cj.ro (L.B.-T.)

**Keywords:** ab initio calculations, half-Heusler materials, magnetic properties, transport properties, spintronics

## Abstract

This study investigates the electronic, magnetic, and transport properties of the Co_0.5_Mn_1.5_Al half-Heusler alloy, a promising candidate for spintronic applications due to its potential half-metallic and ferrimagnetic characteristics. Experimental efforts focus on structural characterization using X-ray diffraction to examine substitutional disorder, such as Co/Mn site migration and Mn/Al site mixing, and their impacts on magnetic and transport properties. Magnetic characterization, including magnetization and susceptibility, reveals an N-type ferrimagnetic behaviour with a Curie temperature of 670 K. Transport experiments probe resistance and magnetoresistance across various temperatures and magnetic fields to uncover conduction mechanisms and spin-dependent effects. Theoretical band structure calculations, utilizing the Korringa–Kohn–Rostoker Green’s function method, investigate the electronic structure and the role of disorder in shaping magnetic and transport properties. This integrated experimental and theoretical approach aims to clarify the alloy’s suitability for applications in exchange bias or antiferromagnetic spintronics.

## 1. Introduction

Since their introduction by de Groot in 1984 [1], half-metallic materials have received significant attention for their potential in spintronics due to their unique electronic structure, exhibiting metallic behaviour in one spin channel and a bandgap at the Fermi level in the other, enabling highly spin-polarized currents [2,3]. While most half-metallic Heusler alloys are ferromagnetic, recent research has focused on half-metallic ferrimagnets (HMFis), which feature inequivalent magnetic sublattices with antiparallel spins, distinguishing them from antiferromagnets by their distinct atomic compositions [4,5,6]. In fully compensated ferrimagnets, a magnetic compensation state with zero net magnetization is achieved, yet the material retains different densities of states for majority and minority spins, offering reduced sensitivity to external magnetic fields and lower energy losses compared to traditional antiferromagnets.

Theoretical and experimental studies have identified several half-metallic ferrimagnets, though many suffer from low Curie temperatures or unstable crystal structures, limiting their technological viability. Notable exceptions include certain Heusler alloys, such as Mn_1.5_V_0.5_FeAl and Mn_2_V_0.5_Co_0.5_Al, which exhibit promising magnetic properties [7,8,9]. The Slater–Pauling rule demands that the total magnetic moment in half-metals should be an integer, requiring specific valence electron counts for full compensation in full-Heusler alloys, often necessitating compositional adjustments [10,11]. The half-Heusler compound Co_0.5_Mn_1.5_Al, with a cubic C1_b_ structure, has been identified as a candidate for near-compensated ferrimagnetic behaviour, potentially suitable for spintronic applications [12]. However, Heusler compounds are prone to antisite disorder, which can significantly influence their electronic, magnetic, and transport properties [3,4].

This study aims to investigate the effects of substitutional disorder on the electronic structure, magnetic behaviour, and transport properties of the Co_0.5_Mn_1.5_Al half-Heusler alloy. Experimental efforts focus on synthesizing the alloy and characterizing its structure through X-ray diffraction to assess disorder. Magnetic measurements, including magnetization and susceptibility, examine the magnetic characteristics and their temperature-dependent variations. Transport experiments examine resistance and magnetoresistance across various temperatures and magnetic fields to elucidate conduction mechanisms and spin-dependent effects. Theoretical band structure calculations, using the Korringa–Kohn–Rostoker Green’s function method, probe the electronic structure and the impact of disorder on magnetic and transport properties, aiming to clarify the alloy’s potential for spintronic applications, such as exchange bias or antiferromagnetic spintronics.

## 2. Materials and Methods

The half-Heusler compound Co_0.5_Mn_1.5_Al was synthetized by induction melting from stoichiometric amounts of 99.9% high-purity elements of cobalt and aluminum. To compensate for the losses caused by evaporation at high temperatures, an extra 4 wt% of manganese was added to the initial composition. The melting process was carried out in a high-purity argon atmosphere. The ingot was turned and remelted four times, in order to ensure the homogeneous mixing of the elements.

Differential Scanning Calorimetry (DSC) was performed using a TA instruments Q600 (TA Instrument, New Castle, DE, USA). Measurements on the as-cast sample were conducted in a pure argon atmosphere in a temperature range of 200 °C to 850 °C. The heating rate was set at 20 °C/min. Two subsequent runs were made on the same piece of sample.

Heat treatments were performed at 500 °C and 600 °C. The heat treatments on the as-cast Co_0.5_Mn_1.5_Al samples were carried out for 48 h in a high vacuum. After treatment, the samples were left to cool naturally.

The crystal structure of the alloys was investigated at room temperature by using a Brüker D8 Advance X-ray diffractometer (Ettlingen, Germany) with Cu K_α_ radiation. The Rietveld refinement of the XRD patterns was performed using the FULLPROF suite [13].

Scanning electron microscopy was carried out on irregular pieces using a Hitachi SU8230 (Tokio, Japan) equipped with an energy-dispersive X-ray spectrometer (EDS) produced by Oxford Instruments (Abingdon, Oxfordshire, UK). Meanwhile, for optical microscopy, the samples were sanded with very fine sandpaper and then slightly etched in a solution of hydrochloric acid until the grain boundaries were revealed.

Magnetization measurements were performed at 4 K using a 7 T vibrating sample magnetometer produced by Cryogenic (London, UK). AC susceptibility was measured using an Oxford Instruments Maglab2000 magnetometer, cooled down to liquid nitrogen temperatures. The measurements were carried out with no applied DC field. The AC field was at a frequency of 1000 Hz and an amplitude of 5 Oe. Thermomagnetic measurements (DC susceptibility) were carried out using a Weiss-type magnetic balance.

Electric measurements were carried out using the four-point probe method, inside a cryostat produced by Cryogenic (7 T) at applied fields of 0, 1, and 2 T and in a temperature range of 10–290 K. The bandgap of the sample was determined by the use of the Arrhenius plot, which is a linearized plot for the conductivity, σ, of semiconductors:

(1) $$\ln\left(\sigma \right)=\ln\left(\sigma_{0}\right)-\frac{E}{k_{B}}\left(\frac{1}{T}\right)$$

where σ is the conductivity, *E* is the energy of the gap, *k_B_* is the Boltzmann constant, and *T* is temperature. Since we only had resistance and not the resistivity value, we used conductance *G* instead of conductivity *σ*. However, since the two quantities are related by a constant, the substitution should only alter the magnitude of *G*_0_ (since it would naturally be $\sigma_{0}$), which was not used in this analysis.

The electronic band structure calculations for the Co_0.5_Mn_1.5_Al Heusler alloy were performed using Density Functional Theory and the spin-polarized relativistic Korringa–Kohn–Rostoker (SPRKKR) method [14]. The computational approach relies on the KKR–Green’s function formalism, which employs multiple scattering theory. The computation method is discussed in more comprehensive detail elsewhere [15]. The fully relativistic mode was employed for the calculations, i.e., all relativistic effects were taken into account, including spin–orbit coupling. The angular momentum expansion of the basis functions was taken up to l = 2. Exchange and correlation effects were calculated using the GGA-PBE parametrization of Perdew et al. [16]. The special points method was used for k-space integration [17]. The substitutional disorder in the system was addressed by means of Coherent Potential Approximation (CPA) [18,19]. Self-consistent band structure calculations were performed using a total energy convergence limit of 10^−6^ Ry.

## 3. Results and Discussion

### 3.1. Structural Characterization

**Figure 1:**
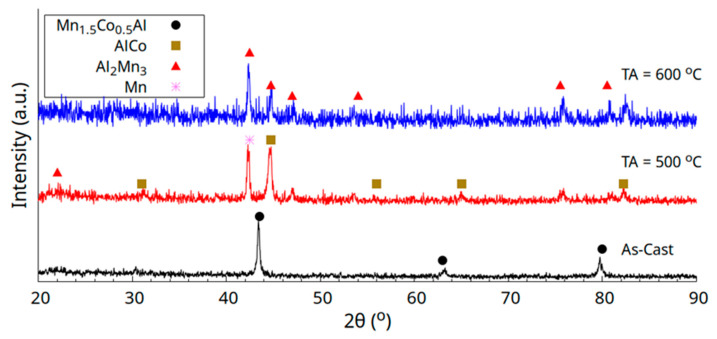
X-ray diffraction patterns of as-cast and thermally annealed Co_0.5_Mn_1.5_Al alloy at 500 °C and 600 °C, respectively.

X-ray diffraction patterns for the as-cast and thermally annealed (TA) samples are given in Figure 1. From the data, we can see that the as-cast sample crystallizes into a cubic structure (just a few peaks are present); however it is disordered (there are no superstructure peaks below 40°), and this will be discussed in further detail later. In an attempt to decrease the atomic antisite disorder in the samples, thermal treatment was carried out at 500 °C and 600 °C. Nevertheless, the diffraction patterns clearly show that annealing reduced the stability of the Heusler phase and caused it to break down into a combination of the AlCo, Mn, and Al_2_Mn_3_ phases. The Al_2_Mn_3_ phase crystallizes in a cubic structure with space group *P4_1_32* (no. 213) and a lattice parameter of a = 6.411 Å, ICDD PDF 00-029-0021 [20]. The AlCo phase adopts a simple cubic structure with space group *Pm-3m* (221) and a lattice constant of a = 2.862 Å, ICDD PDF 00-048-1568, while the Mn phase is tetragonal, with space group *I4/mmm* and lattice parameters a = 2.67 Å and c = 3.55 Å ICDD PDF 00-017-0910.

**Figure 2:**
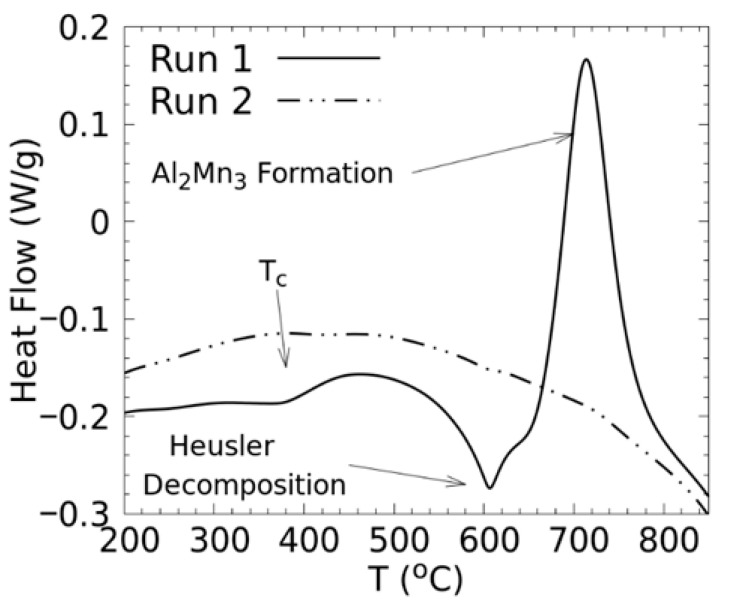
DSC heating curves, two consecutive runs, for the Co_0.5_Mn_1.5_Al alloy.

The decomposition of the Co_0.5_Mn_1.5_Al Heusler phase is also evidenced in the DSC curves (Figure 2). The first run shows a large endothermic peak around 600 °C, which corresponds to the decomposition of the Heusler alloy, and a very large exothermic peak centred around 720 °C which corresponds to the formation of the Al_2_Mn_3_ phase [21]. A small endothermic dip occurs at 400 °C, which corresponds to the magnetic transition, or Curie temperature, of the Heusler alloy. The second run for the Co_0.5_Mn_1.5_Al alloy shows no endothermic or exothermic features, which means that the phase mixtures seen in the XRD patterns are stable.

Given that heat treating the sample leads to the decomposition of the phase of interest, the Co_0.5_Mn_1.5_Al Heusler alloy, into a phase mixture, all subsequent studies are performed on the as-cast sample.

**Figure 3:**
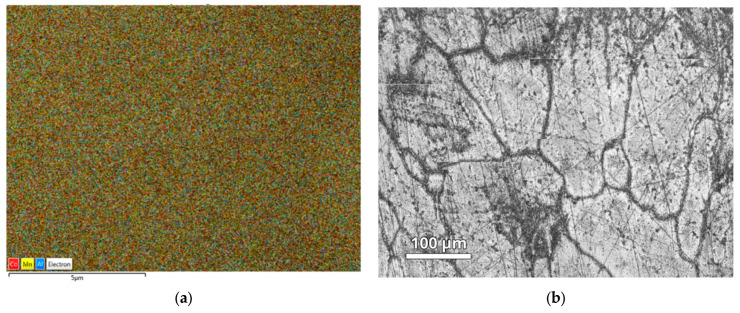
An EDS map at a scale of 5 µm (**a**) and an optical microscopy image at a scale of 100 µm (**b**) for the Co_0.5_Mn_1.5_Al alloy.

The homogeneity of the samples was checked by EDS (Figure 3a), and a good distribution of the Mn, Al, and Co elements was found within the sample. The EDS measurements also showed that the ratio of Mn/Co is within 3% of the desired stoichiometry. The ratio needed to be checked as Mn evaporates during melting, and thus the amount of Mn was uncertain (EDS spectrum given in Appendix A). The microstructure of the sample was also investigated, as shown in Figure 3b. Optical microscopy shows that the grain sizes are large, with most grains being larger than 100 microns in diameter. This observation is important as it means that the microstructure effects, on the magnetic and electric properties, should be rather small.

**Figure 4:**
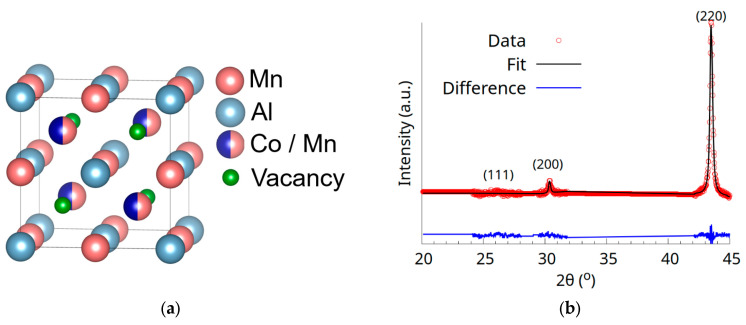
(**a**) The ideal C1_b_ structure of the Co_0.5_Mn_1.5_Al Heusler alloy. (**b**) The XRD patterns and Rietveld refinement of the Co_0.5_Mn_1.5_Al as-cast alloy.

The ideal half-Heusler Co_0.5_Mn_1.5_Al crystallizes in the C1_b_ structure (space group nr. 216). The C1_b_ structure of the Co_0.5_Mn_1.5_Al Heusler alloy, shown in Figure 4a, is formed by three interweaved fcc sublattices with *4a* sites occupied by Al, *4b* sites occupied by Mn, and *4c* sites occupied by Co and Mn, according to previous studies [12,22]. The *4d* sites are considered vacant in the C1_b_ structure [3,4]. The mentioned atomic occupation is in accordance with the one corresponding to the so-called α-phase defined first by Harang [23] for Heusler alloys.

In order to determine the degree and type of atomic disorder in the sample, XRD was remeasured, just at the peak positions but for a much longer duration, and Rietveld refinement was performed on the resulting pattern (Figure 4b)**.** The lattice parameter obtained by the refinement of the XRD patterns in the F-43m (216) space group is 5.89(1) Å, in good agreement with the one found in the literature (5.87 Å) [12]. The XRD pattern displays only the (200) superlattice peak, while the (111) peak is fully extinct. The (111) peak disappears as atoms on *4c* and *4d*, as well as on *4a* and *4b* sites are completely mixed. The potential occurrence of B_2_ antisite disorder was suggested by R. Harikrishnan et al., attributing it to the decrease in the intensity of the (111) and (200) superlattice reflections [12].

The Co_0.5_Mn_1.5_ Al half-Heusler compound is described by the general formula XYZ, with the most electronegative transition metal on X (Co/Mn), the less electronegative transition metal on Y (Mn), and the main group element on Z (Al) [4]. The structure factors were determined for the main three types of reflections, including the order-dependent superlattice peaks (111) and (200) and the (220) reflection that is independent of disorder:

(2) $$F\left(111\right)=4\left(f_{Z}-f_{Y}\right)+4i\;\left(f_{Vc}-f_{X}\right)$$

(3) $$F\left(200\right)=4(f_{Z}+f_{Y}-f_{X}-f_{Vc})$$

(4) $$F\left(220\right)=4\;\left(f_{X}+f_{Y}+f_{Z}+f_{Vc\;}\right)$$

Here, the average atomic scattering factors are considered in the ordered *C1_b_* structure as *f_X_* = (0.5*f_Co_* + 0.5*f_Mn_*); *f_Y_* = *f_Mn_*; and *f_Z_* = *f_Al_*, corresponding to the atomic ordering in the α-phase. The scattering factor of the vacancies is considered to be *f_Vc_* = 0, that is, if the experimentally obtained compound indeed adopts the structure of the α-phase. It is clear that F (111) and F (200) are order-dependent, as they contain difference terms, while the (220) reflection is not affected by substitutional disorder, similarly to the case of full-Heusler alloys described by Webster [24]. Table 1 presents a detailed summary of atomic arrangements for different types of disorders arising from the mixing of atoms on the X, Y, Z, and Vc sites, including combinations such as X-Y, X-Z, Y-Z, Z-Vc, X-Y-Z, X-Vc+Y-Z, and X-Y-Z-Vc. An order parameter S, defined within the range [0, 1], is introduced where S = 0 corresponds to the α-phase for all disorder types, and S increases gradually to S = 1, representing the full statistical mixing of the specified atoms on the crystal sites. The parameter S reflects the proportion of misplaced atoms (X = Co_0.5_Mn_0.5_; Y = Mn; or Z = Al) relative to their positions in the structure of the α-phase.

The disorder types include the Y-Z disorder (B_2_-type) described by the mixing of Al atoms from position *4a* and Mn atoms from position *4b*. Besides these structures, we also studied the case of complete statistical disorder by the mixing of atoms between the *4a*, *4b*, and *4c* sites (W-type disorder, denoted as X-Y-Z). Also, allowing for the occupation of the vacant *4d* sites, the X-Y-Z-Vc disorder type is considered. By applying the calculated atomic site occupancies, we derived the theoretical structure factors |F|^2^ for the order-dependent reflections, namely (111) and (200), using VESTA software (version 3.5.8) [25].

**Figure 5:**
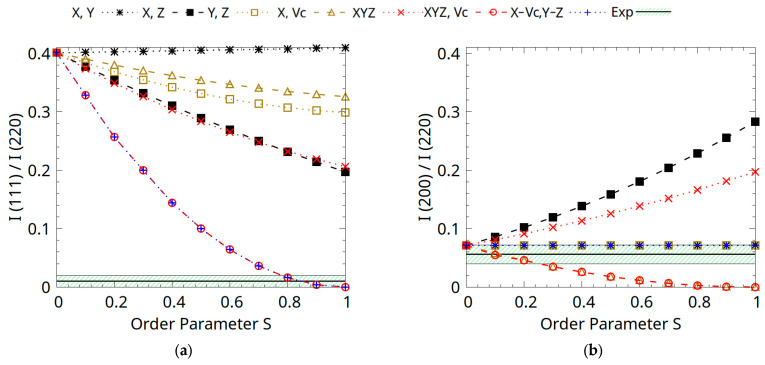
Theoretical powder X-ray diffraction intensity (**a**) I(111)/I(220) and (**b**) I(200)/I(220) plotted as a function of disorder parameter S for the Co_0.5_Mn_1.5_Al alloy. The experimental value is given by a solid black line, with the green marked area around the line representing the uncertainty in the determination.

Given that the intensity of diffraction peaks is directly proportional to the square modulus of the structure factor, this method aims to qualitatively determine the most likely case of ordering by comparing the theoretical and experimental results. To make the experimental and theoretical structure factors directly comparable, excluding the influence of any additional factors, we derived the relative intensities of order-dependent reflections by normalizing all values to the (220) peak intensity. Finally, we represented the evolution of the (111) and (200) relative intensities with the increase in the *S* ordering parameter in Figure 5. These experimentally determined intensity values were also normalized relative to the (220) reflection, unaffected by atomic disorder Equation (4).

The nearly vanishing relative intensity of the (111) peak observed in Figure 4b is a strong indicator of possible disorder types in the Co_0.5_Mn_1.5_Al half-Heusler alloy. This observation is the most consistent with either the X-Y-Z-Vc or X-Vc,Y-Z disorder type, both leading to a significant suppression of the (111) peak with a zero relative intensity for full atomic mixing. This behaviour is supported by Equation (2) which shows that the (111) structure factor depends on the difference between two sets of atomic scattering factors, particularly between *f_Z_* and *f_Y_* and between *f_X_* and *f_Vc_*. We can see in Figure 5a that the X-Y type does not significantly alter the intensity, since Co and Mn, comprising sites X and Y, respectively, have similar scattering factors. In contrast, types X-Z, Y-Z, and X-Y-Z induce noticeable intensity reductions due to mixing between atoms of different atomic numbers and scattering factors, e.g., Al vs. Mn or Co, also by having contributions with different signs in F(111). However, none of this led to the extinction of the (111) reflection. Notably, while a homogeneous mixing involving all atomic and vacant sites (X-Y-Z-Vc) would theoretically yield zero (111) intensity, it would also substantially alter the (200) peak (Figure 5b), which is not observed experimentally. Therefore, the most plausible explanation for the nearly vanishing (111) intensity is a selective mixing between the X-Vc and Y-Z sites, a result also sustained analytically by the expression of the F (111) structure factor.

In the case of the (200) reflection, the experimental value lies within the theoretical prediction for the X-Vc,Y-Z type (Figure 5b), further supporting this disorder model. This consistency arises from the expression of the F(200) structure factor (Equation (3)) where the relevant atomic scattering factors enter with the same algebraic sign (X-Vc and Y-Z). Consequently, even if mixing occurs, it does not significantly alter the intensity, as the net contributions remain balanced. In contrast, other disorder types, such as X-Z and X-Y-Z, lead to a notable increase in the (200) reflection intensity. This behaviour can be attributed to the substitution of atoms with bigger atomic numbers, like Co or Mn (X atoms with larger scattering factors), onto sites initially occupied by atoms with a smaller scattering factor, such as Al or Mn (Z or Y), but where their contribution in the structure factor changes from negative to positive. This results in a constructive effect on the diffraction intensity.

On the other hand, a decrease in the (200) intensity is observed for the Z-Vc and X-Y-Z-Vc disorder types, resulting in nearly overlapping trends with the increase in the disorder parameter *S*. In these cases, the redistribution of atoms with lower scattering power, or the inclusion of vacancies, disrupts the constructive interference, reducing the overall structure factor magnitude. Notably, the Y-Z-type disorder yields a negligible impact on the (200) intensity, as both *f_Y_* and *f_Z_* are in the additive form in F(200), and their interchange has no visible effect on the relative intensities. The same trend is observed for the X-Y-type disorder. Although the F(200) structure factor contains the difference between the two atomic scattering factors, as Mn from the *4b* site and Mn/Co atoms from the *4c* site have similar scattering factors, X-Y mixing does not yield any significant changes in the I(200) intensity.

As a result, an overlap between X-Y-, Y-Z-, and X-Vc+Y-Z-type disorder curves is observed in Figure 5b, and while this might suggest that there are several possible disorder types that would describe the experimental behaviour of the (200) reflection, the only one that is consistent with our results from the (111) peak analysis is the X-Vc,Y-Z type. The atomic configuration of the experimentally synthesized Co_0.5_Mn_1.5_Al sample is optimally characterized by an X-Vc,Y-Z-type disorder. In this arrangement, Mn and Al atoms exhibit mixing between the equivalent *4a* and *4b* crystallographic sites, while a fraction of Mn and Co atoms migrates to the vacant *4d* sites, resulting in the distribution of Mn, Co, and vacancies at the *4c* sites. This atomic configuration aligns closely with neutron diffraction refinement data, with the exception that the neutron diffraction results indicate that Co atoms exclusively occupy the *4c* sites [12].

### 3.2. Magnetic Measurements

**Figure 6:**
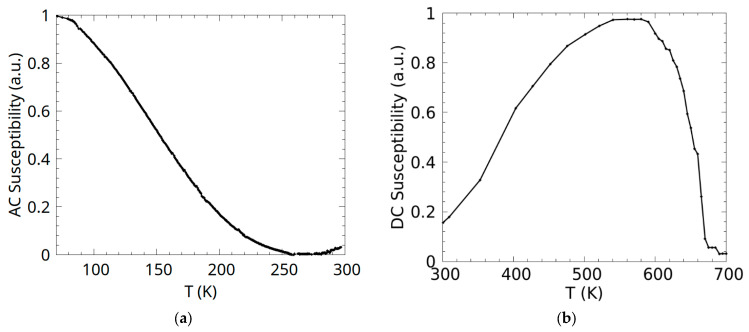
AC susceptibility (**a**) and DC susceptibility (magnetization) (**b**) as a function of temperature for the intermetallic Co_0.5_Mn_1.5_Al compound.

AC susceptibility and magnetization (DC susceptibility) as a function of temperature for the Co_0.5_Mn_1.5_Al sample are given in Figure 6. The sample exhibits characteristic ferrimagnetic properties, showing the asymptotic decrease in susceptibility near the compensation region of the magnetization. The thermomagnetic curves suggest an N-type ferrimagnetic behaviour of the Co_0.5_Mn_1.5_Al compound with the compensation temperature of approximately 260 K (Figure 6a), meaning that in this alloy, the magnetic moments corresponding to one sublattice compensate for the magnetic moments of the other sublattice at a temperature, *T_comp_*, that is well below *T_c_* in this case. The value of the Curie temperature, as can be seen in Figure 6b, was found to be 670 K, which is higher than the one found by Harikrishnan R. et al. (634 K) [12]. Appendix B details the reproducibility test outcomes for the AC and DC measurements of our samples.

**Figure 7:**
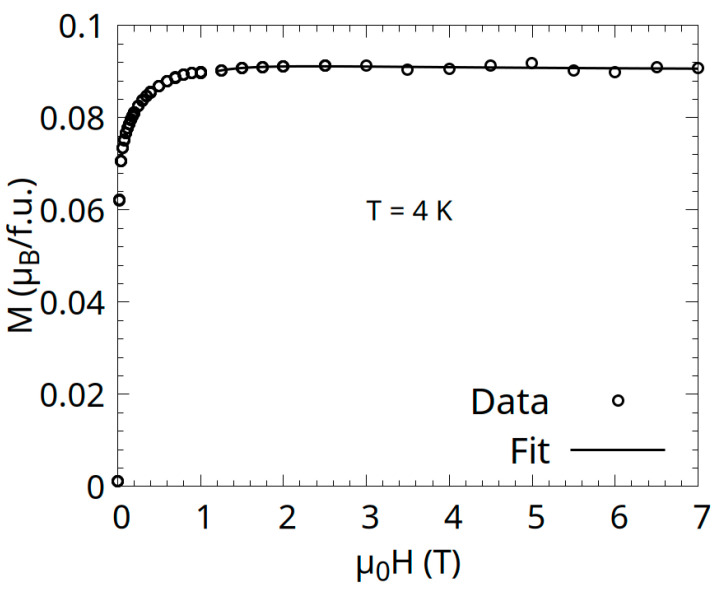
Magnetization as a function of the applied field, at 4 K, for the intermetallic Co_0.5_Mn_1.5_Al compound.

The magnetization curve measured at 4 K is shown in Figure 7. Spontaneous magnetization was determined to be 0.09 $\mu_{B}$/f.u. for our sample, which is in complete agreement with the one found in the literature (0.09 $\mu_{B}$/f.u—[12]).

### 3.3. Transport Measurements

**Figure 8:**
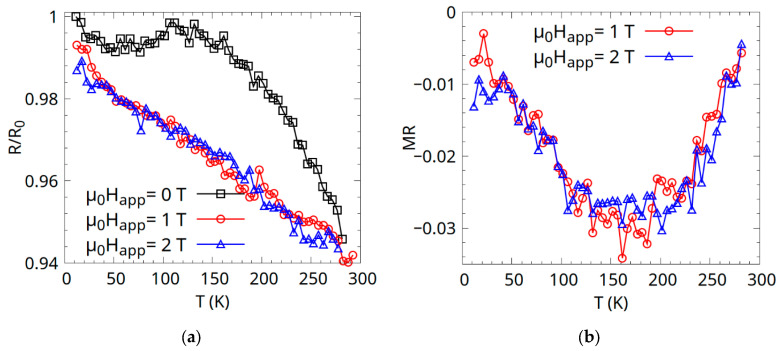
(**a**) Resistance measurements in an applied field of 0, 1, and 2 T as a function of temperature and (**b**) magnetoresistance as a function of temperature, for applied fields of µ_0_H_app_ = 1 and 2 T.

Resistance and magnetoresistance measurements were also carried out as a function of temperature, and the results are given in Figure 8. The values of the resistance are normalized to the value *R*_0_, which is the value of the resistance in the µ_0_H_app_ = 0 applied field, at low temperature.

From the resistance values (Figure 8a), we can see a complex behaviour at lower temperatures, with a decrease with temperature up to 50 K, suggesting increased carrier concentration or mobility. This negative *dR/dT* behaviour can be attributed to strongly disordered metals with an electron mean free path of the order of atomic distances, as described for nanocrystalline Cu_2_MnAl and Co_2_MnSi Heusler alloys [26].

Between 50 and 150 K, a slight increase in resistance could indicate half-metallic behaviour, where the metallic conduction in one spin channel (majority spin) dominates. In half-metals, the majority of spin channels are metallic, and resistance increases with temperature due to electron–phonon and magnon scattering. For the applied field µ_0_H_app_ = 0, at temperatures over 150 K, the magnitude of the resistance drops with temperature, which would indicate a semiconducting behaviour.

When a magnetic field is applied, the shape of the R(T) curve becomes linear and has a (slightly larger) negative slope. This suggests that the magnetic field enhances carrier excitation or mobility in the gapless or semiconducting channel, suppressing scattering mechanisms (e.g., magnon or spin disorder scattering). Linearity may arise from a near-constant density of states at the Fermi level, typical of spin gapless semiconductors (SGSs) or semimetals, where carrier concentration increases linearly with temperature.

The magnetoresistance $MR=\;\frac{R(H)-R(0)}{R(0)}$ × 100% shown in Figure 8b is negative (with a minimum around 150 K), typical of spin-polarized samples which are polycrystalline [27,28,29]. Negative MR arises when the magnetic field aligns spins, reducing spin disorder scattering [30,31] or enhancing conduction in the spin-polarized channel. The minimum at 150 K suggests a peak in spin-dependent scattering at this temperature, possibly due to magnetic phase transitions or magnon activity. The two magnetoresistance curves overlap, as the sample’s magnetization saturates at 1 T, as shown in Figure 7.

**Figure 9:**
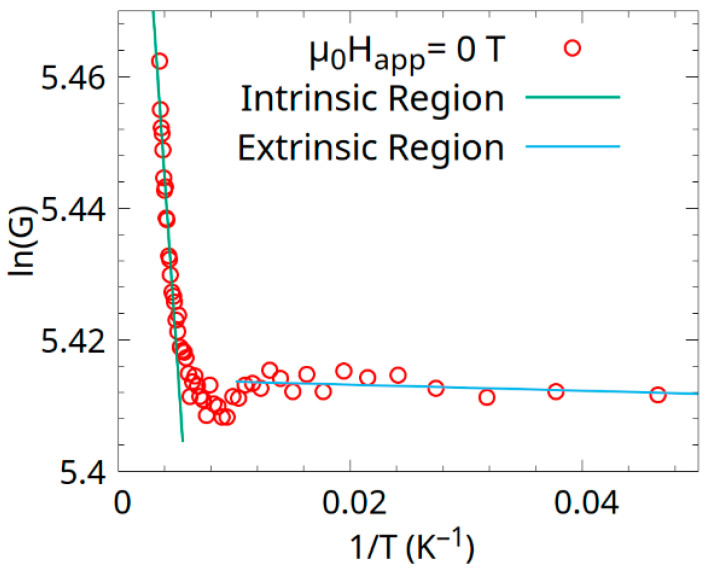
An Arrhenius plot showing the logarithmic conductance as a function of reciprocal temperature. The linear fits in the two regions are also displayed.

The Arrhenius plot was constructed relative to the conductance of the sample G, Figure 9, and the corresponding energy gaps were found. The linear fits revealed a possible activation energy of 0.006(4) meV for the extrinsic region and an activation energy of 3.2(2) meV for the intrinsic region. In the extrinsic region (low T), the small gap enables easy thermal excitation, contributing to the observed resistance decrease from 5 to 50 K in the zero field. The polycrystalline nature may introduce defect states, narrowing the effective gap. In the intrinsic region (high T), an activation energy of 3.2 ± 0.2 meV indicates intrinsic two-carrier conductivity, involving both electrons and holes, typical of a narrow-gap or gapless semiconductor at higher temperatures [32] (e.g., >150 K, where R/R_0_ decreases slightly). Over 150–300 K (k_B_T ≈ 12.9–25.9 meV), thermal energy easily excites carriers across the 3.2 meV gap, increasing conductance and reducing resistance, consistent with the negative temperature coefficient in Figure 8.

According to the Arrhenius plot, the band structure exhibits small activation energies for both carrier types, likely due to overlapping or closely spaced conduction and valence bands. Overlapping conduction and valence bands could produce electron and hole pockets, typical of semimetals (e.g., Bi). The small gaps and linear dependence of resistivity under field support this, with high carrier density and spin-polarized transport due to the Heusler alloy’s magnetic nature.

### 3.4. Theoretical Calculations

**Figure 10:**
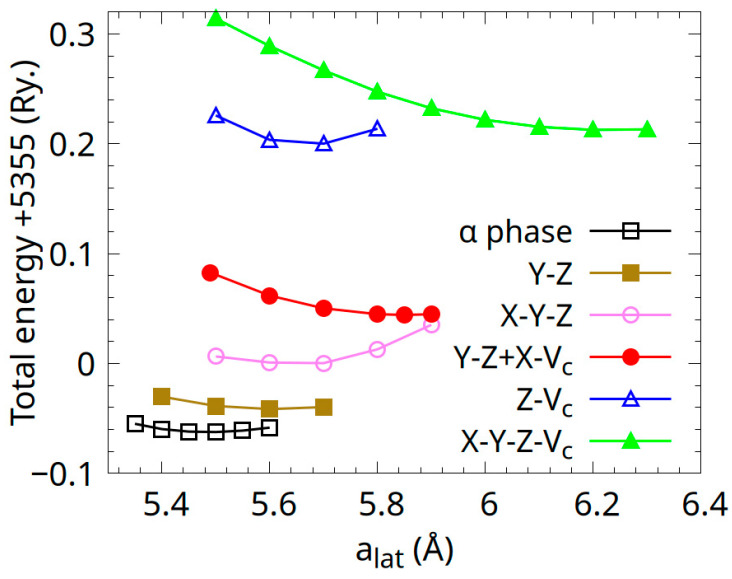
The total energy per formula unit vs. lattice constant *a* for the Co_0.5_Mn_1.5_Al Heusler alloys with disorder types described in Table 1 for full atomic mixing (S = 1).

Band structure calculations for the Co_0.5_Mn_1.5_Al Heusler alloy considering the C1_b_ structure (space group nr. 216) and the disorder types described in Table 1 were performed for S = 1. The total energies plotted as a function of lattice constant *a* are shown in Figure 10. As can be seen in Figure 10, the α-phase is the most stable. The Y-Z disorder type system is higher in energy with ΔE = 23.6 meV/atom, close to the thermic energy of 26 meV/atom. The W-type X-Y-Z disordered system exhibits higher energy with ΔE = 72.5 meV/atom compared to the α-phase. According to the total energy calculations, the occupation of the *4d* site with mixed atom types is less favourable. Between these types of systems, by considering the mixing of atoms between Y-Z and X-Vc, the total energy increases by 120.8 meV/atom compared with that of the α-phase. All other considered cases, including mixing and the *4d* site occupation, are less favourable, as shown in Table 2.

The theoretical lattice constants obtained for all investigated systems are listed in Table 2. As can be seen, the lattice constant of the α-phase is 5.5 Å, which is about 6% lower than the experimental value. The Co_0.5_Mn_1.5_Al Heusler alloy is derived from CoMnAl through the addition of extra manganese. Accounting for the greater metallic radii of Mn than that of Co atoms, the theoretical lattice constant of Co_0.5_Mn_1.5_Al is consistent with that of the ferrimagnetic phase of the CoMnAl alloy (*a* = 5.46 Å) [22].

**Figure 11:**
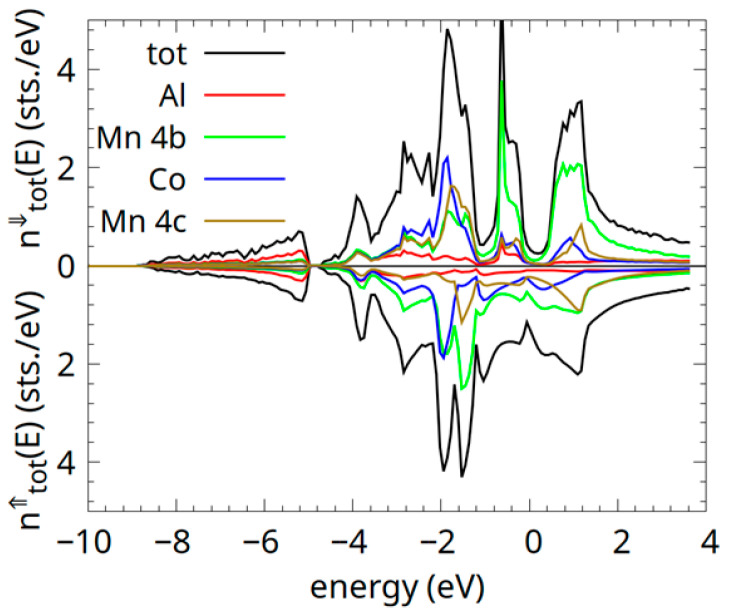
The spin-resolved density of states (DOS) calculations for the α-phase of the Co_0.5_Mn_1.5_Al alloy. The Al (red), Mn *4b* (light green), Co (blue), and Mn *4c* (dark green) type-resolved DOS are shown.

The significant discrepancy between theoretical and experimental lattice constants in Co_0.5_Mn_1.5_Al Heusler alloy samples suggests structural disorder, likely arising from thermal activation, non-equilibrium synthesis conditions, or off-stoichiometry. XRD analysis (Section 3.1) confirms the presence of disorder, such as antisite defects in these samples. Additionally, the wide range of equilibrium lattice constants observed in disordered systems (Table 1) indicates further contributions from defects like interstitials or vacancies, which may account for the substantial lattice parameter deviations. The density of states (DOS) calculations for the α-phase Co_0.5_Mn_1.5_Al are shown in Figure 11.

In Co_0.5_Mn_1.5_Al, the excess Mn leads to a density of states (DOS) with overlapping spin-up and spin-down contributions near the Fermi level, primarily driven by Mn *3d* states. A small DOS persists in the spin-down channel’s quasi-bandgap, reducing spin polarization due to Mn *3d-3d* hybridization. Additionally, a dip in the DOS is observed at the Fermi level in the spin-up channel. This results in a ferrimagnetic state with a low magnetic moment. Nevertheless, Co_0.5_Mn_1.5_Al retains significant spin polarization (~70%).

**Figure 12:**
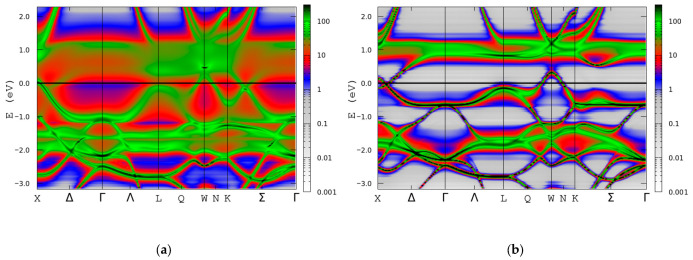
Bloch spectral functions (BSFs) for spin-up (**a**) and spin-down (**b**) channels in Co_0.5_Mn_1.5_Al alloy. Bloch spectral function (BSF) intensity is represented by colour scheme adjacent to each figure.

In order to investigate the half-metallic character, Bloch spectral functions are plotted for both the spin-up and spin-down channels in Co_0.5_Mn_1.5_Al alloys (Figure 12). Band crossing (near the X and W points in the Brillouin zone) in the spin-down channel at the Fermi level is present for both alloys, suggesting a spin semimetallic (SSM) electronic behaviour.

The magnetic moments calculated for the Co_0.5_Mn_1.5_Al Heusler alloy by accounting for the general gradient approximation (GGA) for the exchange and correlation effects are shown in Table 3.

In CoMnAl [22], the ferrimagnetic ground state consists of an antiparallel spin arrangement of Mn *4b* (1.38 μ_B_), Co *4c* (−0.25 μ_B_), and Al *4a* (−0.1 μ_B_) spin moments. The AFM or ferrimagnetic order arises from direct Mn *3d*-*3d* hybridization, which competes with Co-Mn and Mn *3d*-Al *sp* hybridization.

According to the Bethe–Slater curve [33], the Mn-Mn exchange interactions tend to be antiferromagnetic (antiparallel) when Mn atoms are close (first nearest neighbours) and may become ferromagnetic at larger distances. The excess of Mn in Co_0.5_Mn_1.5_Al introduces antiferromagnetic Mn *4b*-Mn *4c* coupling at shorter distances ($\sqrt{3}$/4 *a*). Partial antiferromagnetic Mn *4b*-Mn *4c* coupling reduces the net magnetic moment and closes the half-metallic gap in Co_0.5_Mn_1.5_Al.

The site-dependent magnetic moments obtained from the band structure calculations confirm the ferrimagnetic ground state of the Co_0.5_Mn_1.5_Al Heusler alloy. According to our GGA calculations, the Co and Mn on site *4c* are ferromagnetically coupled, with magnetic spin moments of −0.32 μ_B_ for Co and −1.53 μ_B_ for Mn. Their negative sign shows the antiparallel alignment between Mn/Co *4c* and Mn *4b* spins. The spin compensation is not complete, and a net spin magnetic moment (0.11 μ_B_) is obtained. One has to mention that the quasi-compensated ferrimagnetic behaviour is maintained for a rather large range of lattice constant values (5.35–5.75 Å). This magnetic behaviour is in line with the experimental measurements (0.09 μ_B_/f.u.). Also, the missing half-metallic character of the Co_0.5_Mn_1.5_Al Heusler alloy explains this slight uncompensated spin moment. The magnetic moments calculated by the GGA approach agree well with the values reported by Ma et al. for the CoMnAl Heusler alloy (1.38 μ_B_ for Mn *4b* and −0.25 μ_B_ for Co *4c* [22].

### 3.5. Disorder Effects

The effects of disorder on the magnetic properties of the Co_0.5_Mn_1.5_Al Heusler alloy were investigated. Magnetic moments, calculated using the GGA approach for disordered configurations with total energies up to 150 meV/atom above the α-phase (Figure 10), are reported in Table 3.

The magnetic moment of disordered states increases proportionally to the energy required to form these states relative to the α-phase. The magnetic spin moment of Mn on the *4c* site remains antiparallel to Mn on the *4b* site in all cases, consistent with the α-phase. In disordered systems, Mn and Co spins at the *4c* site display ferrimagnetic coupling, in contrast to their parallel ferromagnetic alignment in the ordered α-phase. This ferrimagnetic coupling is the primary cause of the increased magnetic moment in these disordered systems. Increased moments in disordered states reflect reduced cancellation due to antisite defects (Mn *4a*, Al *4b*, Co *4a*, *4b*, *4d*) disrupting the α-phase’s balanced spin structure.

As seen in structural characterization, the samples are disordered, and the most probable substitutional disorder is due to the Co and Mn atoms from *4c* sites migrating to the vacant *4d* sites, creating Co *4d* and Mn *4d* antisites and leaving vacancies at the *4c* site. Co vacancies at *4c* weaken Co-Mn hybridization, further stabilizing a semimetal state with electron and hole pockets.

In addition, Mn (normally on *4b*) occupies *4a* Al sites, creating Mn *4a* antisites, and Al (normally on *4a*) occupies *4b* (Mn sites, Al *4b* antisites), causing significant site disorder. Disorder from Co/Mn *4d*, Mn *4a*, and Al *4b* antisites smears the DOS, enhancing band overlap and closing any bandgap, as can be seen in Appendix C. This analysis supports a gapless or near-gapless semimetal, enabling easy carrier excitation.

Antisites and vacancies introduce localized states near the Fermi level, contributing to the 0.006 meV gap (extrinsic conduction) and modulating the 3.2 meV gap (intrinsic two-carrier conduction). The 0.006 meV gap reflects impurity-induced conduction from defect states (Co *4d*, Mn *4d*, Mn *4a* vacancies). These states act as shallow donors/acceptors, increasing carrier concentration (n_e_, n_h_) with minimal thermal energy (*k_B_T* ≈ 0.43 meV at 5 K), driving the 5–50 K resistance decrease. The 3.2 meV gap reflects intrinsic two-carrier conduction, where band overlap allows for the thermal excitation of electrons and holes (*k_B_T* ≈ 12.9–25.9 meV). Disorder enhances the DOS at the Fermi level (as seen in Appendix C), driving the negative temperature coefficient (slight resistance decrease above 150 K). Previous data show similar gaps (3 meV electrons, 0.3 meV holes) [12], suggesting a comparable semimetal structure, but a smaller 0.006 meV gap indicates stronger disorder effects. The polycrystalline nature introduces grain boundaries, amplifying disorder effects.

Antisite defects and vacancies create localized spin misalignment, increasing resistivity in the zero field. The field reduces this, enhancing conductivity and producing negative MR, as seen in polycrystalline samples [27]. From the resistance measurements at µ_0_H_app_ = 1 T, it can be seen that magnetic field suppresses spin disorder and eventual magnon scattering, increasing mobility and leading to the linear R(T) curve with a steeper negative slope. The high DOS from disorder supports linear carrier excitation, reducing resistance.

## 4. Conclusions

The Co_0.5_Mn_1.5_Al half-Heusler alloy adopts a cubic C1_b_ structure. X-ray diffraction analysis, combined with form factor analysis for various disorder types, confirms X-Vc,Y-Z disorder as the most likely configuration in the experimentally synthesized sample. This disorder involves Co and Mn migration from *4c* to *4d* sites and Mn/Al mixing between *4a* and *4b* sites, leading to antisites and vacancies that disrupt the ideal α-phase structure.

Magnetic measurements reveal ferrimagnetic behaviour with a high Curie temperature and a compensation point well below it, characteristic of N-type ferrimagnetism. The low saturation magnetization observed indicates near-compensated ferrimagnetism. Transport measurements show complex resistance behaviour: a decrease at low temperatures due to defect-driven conduction, an increase at intermediate temperatures, and a slight decrease at higher temperatures, reflecting two-carrier conduction and a negative temperature coefficient. Under a magnetic field, resistance decreases linearly, suggesting suppressed scattering, while negative magnetoresistance highlights spin-polarized transport in polycrystalline samples.

Theoretical calculations confirm the α-phase as the ground state, with low-energy disordered phases. The density of states shows spin semimetal behaviour, driven by overlapping spin-up and spin-down bands, influenced by Mn *3d* states and disorder. In the α-phase, Co and Mn at *4c* are ferromagnetically coupled, with antiferromagnetic Mn *4b*–Mn *4c* coupling reducing the net moment. Disorder increases magnetic moments, reducing compensation. These properties suggest potential for exchange bias or antiferromagnetic spintronic applications.

## Figures and Tables

**Table 1:** The arrangements of atoms of a half-Heusler alloy with the general formula XYZ, on four interpenetrating FCC sublattices if we consider that in the mixing of atoms, the theoretically vacant sublattice is also involved, defining S as the fraction of X = Co_0.5_Mn_0.5_; Y = Mn; or Z = Al atoms that are misplaced in comparison to their position in their structure corresponding to phase α.

Type of Disorder	*4c* (1/4,1/4,1/4)	*4b* (1/2,1/2,1/2)	*4a* (0,0,0)	*4d* (3/4,3/4,3/4)
α-phase	X	Y	Z	Vc
X-Y	$\left(1-\frac{S}{2}\right)X+\frac{S}{2}\;Y$	$\left(1-\frac{S}{2}\right)Y+\frac{S}{2}X$	Z	Vc
X-Z	$\left(1-\frac{S}{2}\right)X+\frac{S}{2}Z$	Y	$\left(1-\frac{S}{2}\right)Z+\frac{S}{2}X$	Vc
Y-Z	X	$\left(1-\frac{S}{2}\right)Y+\frac{S}{2}Z$	$\left(1-\frac{S}{2}\right)Z+\frac{S}{2}Y$	Vc
Z-Vc	X	Y	$\left(1-\frac{S}{2}\right)Z$	$\frac{S}{2}Z$
X-Y-Z	$\left(1-\frac{2}{3}S\right)X+\frac{S}{3}(Y+Z)$	$\frac{S}{3}\left(X+Z\right)+\left(1-\frac{2}{3}S\right)Y$	$\frac{S}{3}\left(X+Y\right)+\left(1-\frac{2}{3}S\right)Z$	Vc
X-Vc,Y-Z	$\left(1-\frac{S}{2}\right)X$	$\left(1-\frac{S}{2}\right)Y+\frac{S}{2}Z$	$\left(1-\frac{S}{2}\right)Z+\frac{S}{2}Y$	$\frac{S}{2}X$
X-Y-Z-Vc	$\left(1-\frac{3}{4}S\right)X+\frac{S}{4}\left(Y+Z\right)$	$\frac{S}{4}(X+Z)+\left(1-\frac{3}{4}S\right)Y$	$\frac{S}{4}\left(X+Y\right)+\left(1-\frac{3}{4}S\right)Z$	$\frac{S}{4}(X+Y+Z)$

**Table 2:** Theoretical lattice constants and energy difference ΔE compared to the most stable (α-phase) in the Co_0.5_Mn_1.5_Al Heusler alloy. Disorder types described by the full mixing (S = 1) of Y-Z, X-Y-Z, Y-Z+X-Vc, Z-Vc, and X-Y-Z-Vc atoms are considered.

Type of Disorder	a_lat_ (Å)	ΔE (meV/at.)
α-phase	5.50	-
Y-Z	5.61	23.6
X-Y-Z	5.63	72.5
Y-Z,X-Vc	5.85	120.8
Z-Vc	5.67	297
X-Y-Z-Vc	6.20	315

**Table 3:** Calculated magnetic moments of disordered Co_0.5_Mn_1.5_Al Heusler alloy with order parameter S = 1 by means of GGA approach. Spin (m_s_) and orbital (m_l_) magnetic moments are expressed in Bohr magnetons.

		α-Phase		Y-Z		X-Y-Z		Y-Z+X-Vc	
		m_s_ (μ_B_)	m_l_ (μ_B_)	m_s_ (μ_B_)	m_l_ (μ_B_)	m_s_ (μ_B_)	m_l_ (μ_B_)	m_s_ (μ_B_)	m_l_ (μ_B_)
*4a*	Co	-	-	-	-	0.87	0.08	-	-
	Mn	-	-	2.73	0.02	2.79	0.02	3.81	0.01
	Al	−0.06	-	−0.02	-	−0.02	-	−0.01	-
*4b*	Co	-	-	-	-	0.85	0.08	-	-
	Mn	1.10	0	2.73	0.02	2.78	0.02	3.81	0.01
	Al	-	-	−0.02	-	−0.02	-	−0.01	-
*4c*	Co	−0.32	−0.02	0.46	0.01	1.04	0.04	0.56	0.02
	Mn	−1.53	−0.02	−2.12	−0.02	−2.41	−0.02	−1.84	−0.03
	Al	-	-	-	-	−0.12	-	-	-
*4d*	Co	-	-	-	-	-	-	0.56	0.02
	Mn	-	-	-	-	-	-	−1.84	−0.03
	total	0.11	−0.02	1.90	0.01	2.01	0.04	3.22	0.00

**Figure A1:**
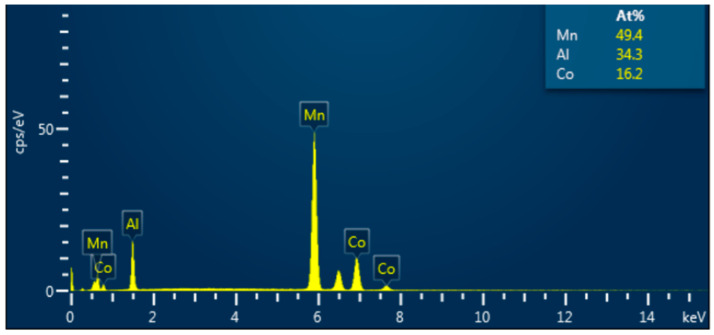
EDX (energy-dispersive X-ray analysis) spectrum for Co_0.5_Mn_1.5_Al as-cast alloy.

**Figure A2:**
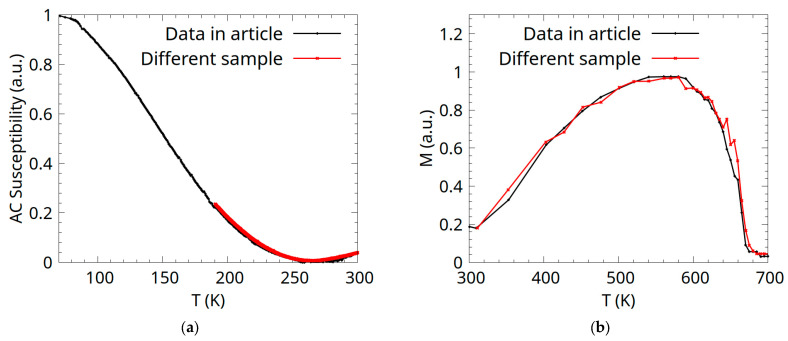
This figure shows low-temperature AC (**a**) and high-temperature DC (**b**) susceptibility for the Mn1.5Co0.5Al alloy. The graphs show the data included in this article in black and data recorded on a different sample in red.

**Figure A3:**
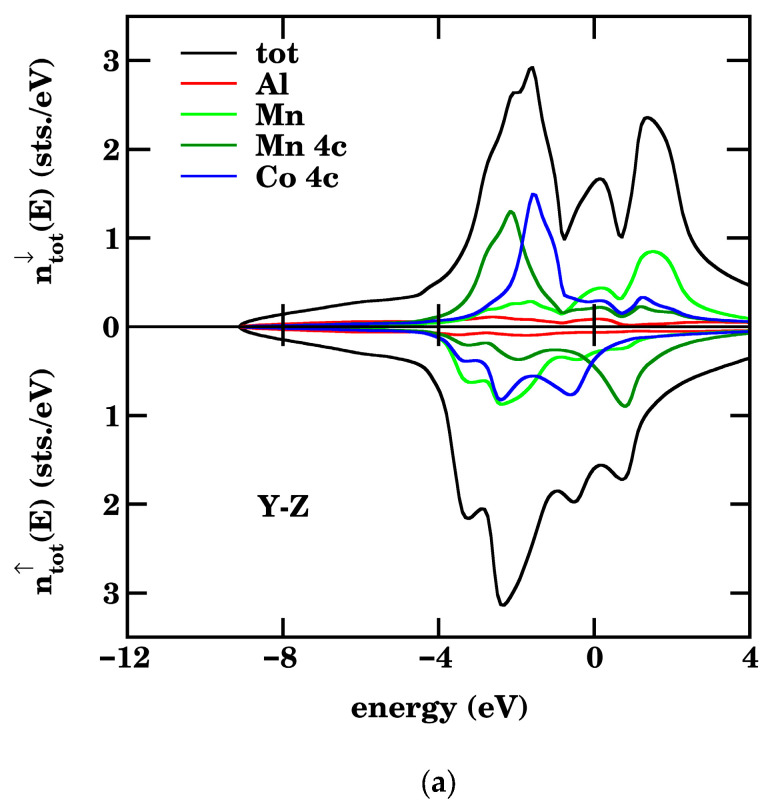

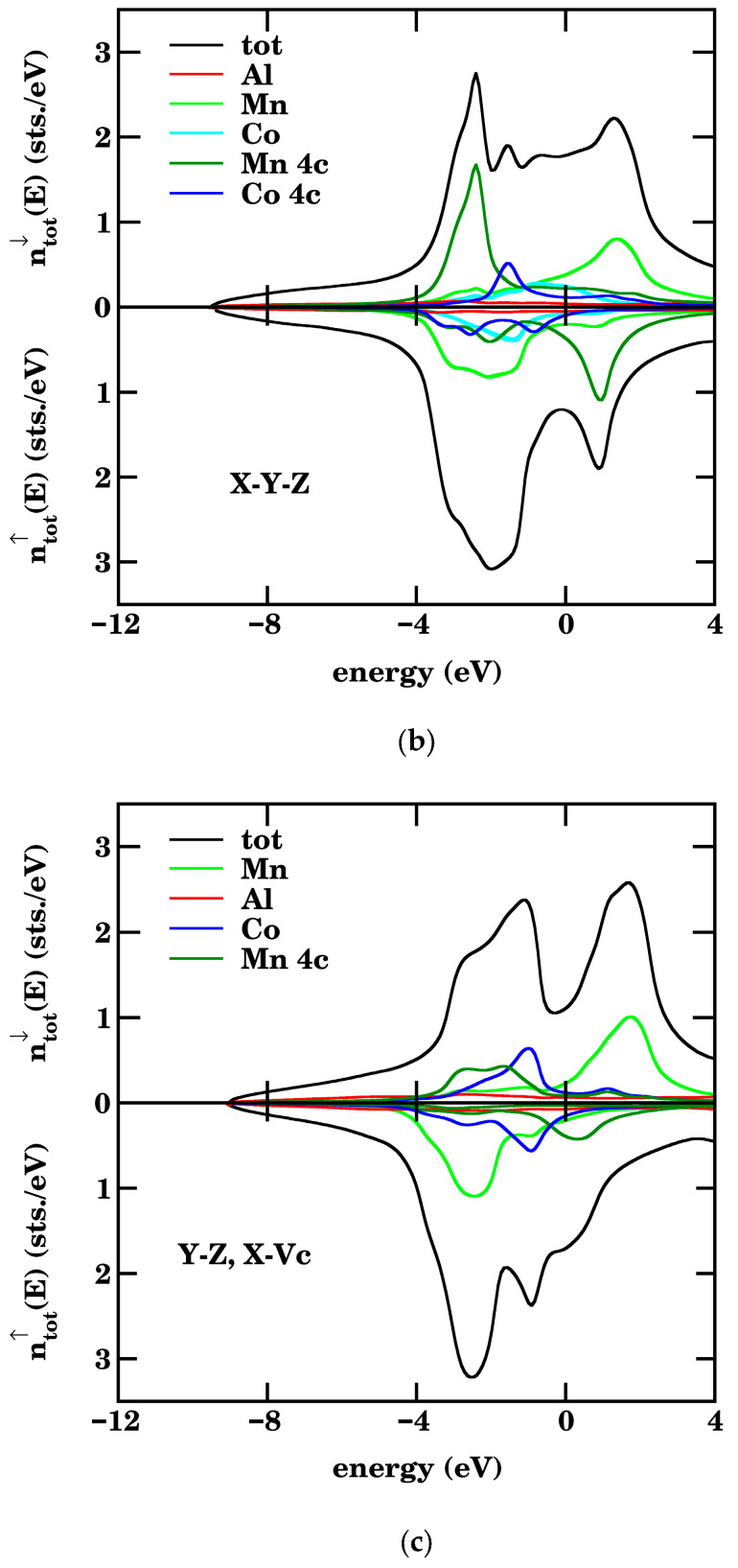
(**a**)The spin-resolved density of states (DOS) for the Co_0.5_Mn_1.5_Al half-Heusler alloy and the cubic C1_b_ structure, with disorder type B_2_ (Y-Z type), (**b**) W-type (X-Y-Z), and (**c**) the X-Vc,Y-Z disorder type suggested by structural analysis.

## Data Availability

Data are available from the corresponding author upon reasonable request.

## Abbreviations

The following abbreviations are used in this manuscript:

DSC	Differential Scanning Calorimetry
CPA	Coherent Potential Approximation
KKR	Korringa–Kohn–Rostoker
HMFi	Half-Metallic Ferrimagnet
XRD	X-Ray Diffraction

## Appendix A

The composition of the as-cast sample needed to be investigated as Mn evaporates during melting. While an excess of Mn was introduced to compensate for evaporation, the exact ratios between the elements were checked. Figure A1 shows that the ratios between the elements are close to the ideal amounts: Mn/Co is off stoichiometry by 2%, while the Al content seems to be off by 3%. However it is reasonable to expect a 1–2% error in the determination, especially for the lower Z elements.
